# Human epidermal resident memory T cells: beyond the dermal perspective

**DOI:** 10.3389/fimmu.2026.1903376

**Published:** 2026-07-16

**Authors:** Youichi Ogawa, Takuya Sato, Lisa Minai, Manao Kinoshita, Shinji Shimada, Tatsuyoshi Kawamura

**Affiliations:** Department of Dermatology, Faculty of Medicine, University of Yamanashi, Yamanashi, Japan

**Keywords:** epidermis, human skin, Langerhans cells, resident regulatory T cells, skin immune homeostasis, tissue-resident memory T cells

## Abstract

The concept of tissue-resident memory T (T_RM_) cells has fundamentally transformed our understanding of peripheral immune surveillance. Although skin T_RM_ biology has been extensively studied, most investigations have focused on the dermis, whereas the epidermis has traditionally been regarded primarily as a physical barrier composed predominantly of keratinocytes. However, accumulating evidence has demonstrated that the normal human epidermis harbors abundant populations of resident T cells, including both conventional and regulatory T_RM_ cells, indicating that the epidermis is a highly specialized immunological compartment. In this review, we summarize current knowledge regarding the origin, composition, phenotype, and dynamics of human epidermal T_RM_ cells, with particular emphasis on studies performed in human tissues. Epidermal T_RM_ cells are enriched for CD8^+^CD69^+^CD103^+^ populations and include specialized cytotoxic subsets expressing CD49a and CD101, whereas regulatory T_RM_ cells constitute a distinct epithelial regulatory network that contributes to local immune tolerance. We also discuss recent advances demonstrating that epidermal T_RM_ cells actively patrol the epidermis, interact closely with Langerhans cells, and participate in the maintenance of epidermal immune homeostasis. Furthermore, emerging evidence implicates epidermal T_RM_ cells in a variety of human diseases, including HIV infection, fixed drug eruption, vitiligo, and alopecia areata. Collectively, these findings support a model in which conventional T_RM_ cells, regulatory T_RM_ cells, Langerhans cells, and keratinocytes form an integrated epidermal immune ecosystem that balances immune surveillance and immune regulation. Recognition of this cellular network challenges the traditional dermis-centric view of cutaneous immunity and suggests that the epidermis should be regarded as an autonomous immunological compartment. A deeper understanding of epidermal immune organization may provide new insights into the pathogenesis and treatment of epidermis-centered diseases.

## Highlights

- The human epidermis harbors abundant populations of conventional and regulatory tissue-resident memory T cells.
- Epidermal T_RM_ cells are enriched for CD8^+^CD69^+^CD103^+^ populations and include specialized CD49a^+^ and CD101^+^ cytotoxic subsets.
- Epidermal immune homeostasis is maintained through interactions among conventional T_RM_ cells, regulatory T_RM_ cells, Langerhans cells, and keratinocytes.
- Epidermal T_RM_ cells contribute to the pathogenesis of HIV infection, fixed drug eruption, vitiligo, and alopecia areata.
- The epidermis should be regarded as an autonomous immunological compartment rather than merely a passive target of dermal inflammation.

## Introduction

Human skin is composed of three major layers: the epidermis, dermis, and subcutaneous tissue. The epidermis is the outermost layer and is composed predominantly of keratinocytes. Beneath the epidermis lies the dermis, a collagen-rich connective tissue layer that is substantially thicker than the epidermis. The dermis contains a diverse population of CD45^+^ immune cells derived from hematopoietic lineage cells (HLCs), including tissue-resident memory T (T_RM_) cells, dendritic cells, macrophages, natural killer cells, innate lymphoid cells, and mast cells. In contrast, the epidermis primarily serves as a barrier against external insults and has long been regarded as a compartment composed predominantly of CD45⁻ keratinocytes, with only minor populations of CD45⁻ melanocytes, Merkel cells, and CD45^+^ Langerhans cells (LCs). The relative paucity of CD45^+^ HLC-derived immune cells in the epidermis has long contributed to the notion that the epidermis is immunologically less dynamic than the dermis (1). However, it is now well established that human T_RM_ cells reside not only in the dermis but also in the epidermis (2). The recognition of abundant T_RM_-cell populations within the epidermis has fundamentally changed our understanding of epidermal immunity and prompted a reassessment of the composition, phenotype, and functional significance of cutaneous T cells. The discovery of epidermal T_RM_ cells has also raised the possibility that LCs, traditionally viewed as antigen-presenting cells that transport external antigens to draining lymph nodes, may additionally regulate local immune responses through direct interactions with epidermal T_RM_ populations.

Importantly, many of the fundamental concepts of T_RM_ biology were originally established in murine models. However, steady-state mouse skin differs markedly from human skin in that it contains relatively few conventional αβ T cells and is instead dominated by γδ T cells, particularly within the epidermis (3). Because robust epidermal T_RM_ populations in mice are typically generated only after infection or inflammatory stimuli, extrapolation of murine findings to human skin requires caution. Therefore, in this review, we focus primarily on studies of human skin and highlight recent advances in our understanding of epidermal T_RM_ cells, including their composition, developmental origins, dynamic behavior, interactions with LCs, and roles in maintaining epidermal immune homeostasis.

Rather than functioning solely as a physical barrier, the human epidermis is increasingly recognized as a highly specialized immunological compartment that harbors distinct populations of conventional and regulatory T_RM_ cells and supports complex cellular interactions essential for tissue immune surveillance, immune regulation, and epithelial homeostasis.

## Discovery of human skin-resident memory T cells

The concept of skin-resident memory T_RM_ cells emerged through a series of studies demonstrating that memory T cells can persist within peripheral tissues independently of continuous replenishment from the circulation. Early evidence came from observations in fixed drug eruption (FDE), in which disease recurrence consistently occurred at the same anatomical site. Mizukawa et al. identified long-lived epidermal CD8^+^ effector-memory T cells at healed FDE lesions and showed that these cells rapidly produced IFN-γ upon drug re-exposure, suggesting that memory T cells could persist locally within the epidermis and mediate site-specific immune responses (4). Boyman et al. subsequently demonstrated that symptomless skin from patients with psoriasis retained the capacity to develop psoriatic lesions after transplantation onto immunodeficient mice, indicating that pathogenic memory T cells could persist locally within clinically normal skin (5). These findings were further extended by Clark et al., who estimated that the skin contains approximately 2 × 10^10^ T cells, nearly twice the number present in the circulation, highlighting the skin as a major reservoir of memory T cells (6).

Following these landmark studies, murine cutaneous viral infection models established the concept of T_RM_ cells. Using a herpes simplex virus (HSV) infection model, Gebhardt et al. demonstrated that non-recirculating memory CD8^+^ T cells persist within previously infected skin and provide local protection against reinfection (7, 8). Supporting these findings in humans, Zhu et al. showed that CD8αα^+^ T cells persist at sites of prior HSV-2 reactivation and exhibit phenotypic and functional characteristics consistent with tissue residency (9). Returning to murine studies, Mackay et al. further defined the developmental pathway and phenotype of skin T_RM_ cells, demonstrating that they sequentially acquire CD69 and CD103 (αEβ7 integrin) expression and require both molecules for optimal tissue residency and long-term persistence, thereby establishing the canonical CD69^+^CD103^+^ T_RM_ phenotype recognized today (10). Jiang et al. subsequently showed that skin infection generates long-lived CD8^+^ T_RM_ cells that populate distant skin sites, extending immune surveillance beyond the original site of infection and supporting the concept of global skin immunity (11). Together, these murine studies established the fundamental principles of T_RM_ biology and provided the conceptual framework for subsequent investigations of human skin T_RM_ cells.

In parallel, advances in human skin research provided direct evidence for resident T-cell populations. Using skin samples from alemtuzumab-treated patients, in whom circulating T cells are profoundly depleted, Clark et al. demonstrated that substantial numbers of memory T cells persist within the skin. This study provided direct evidence that human skin T_RM_ cells can survive independently of the circulating T-cell pool (12). Watanabe et al. subsequently provided a comprehensive characterization of the human skin memory T-cell compartment and established several concepts that continue to shape the field today. Using a human skin-grafted mouse model together with analyses of healthy human skin, the authors demonstrated that human skin contains distinct populations of CD69^+^CD103^+^ and CD69^+^CD103⁻ T_RM_ cells, with CD103^+^ T_RM_ preferentially enriched in the epidermis and CD103⁻ T_RM_ predominating in the dermis. Importantly, these findings revealed a level of heterogeneity not previously appreciated from murine studies, in which T_RM_ cells had been largely defined as CD103^+^ epidermal populations (2). Collectively, these studies transformed our understanding of human cutaneous immunity by establishing the existence, heterogeneity, and tissue organization of skin T_RM_ cells, and by identifying the epidermis as a key anatomical niche for CD103^+^ T_RM_ cells.

## Origin of human skin-resident memory T cells

### Origin of conventional T_RM_ cells

Watanabe et al. proposed a framework in which human skin immunity is maintained by four functionally distinct populations of memory T cells, including two resident populations (CD69^+^CD103^+^ and CD69^+^CD103⁻ T_RM_ cells) and two recirculating populations, namely central memory T cells (T_CM_; CCR7^+^L-selectin^+^) and migratory memory T cells (T_MM_; CCR7^+^L-selectin⁻) (2). This model suggested that recirculating memory T cells may serve as precursors of skin T_RM_ cells following entry into peripheral tissues. Using human skin samples and a human skin-grafted mouse model, Gehad et al. demonstrated that T_CM_ are present in healthy skin and can migrate into peripheral tissues, where they mediate local immune responses. These findings suggested that circulating T_CM_ may contribute directly to tissue immunosurveillance and represent a potential source of skin T_RM_ cells (13). Therefore, using a healthy human skin-grafted NSG mouse model, Matos et al. systematically compared the T_RM_-generating capacity of human circulating memory T-cell subsets and demonstrated that T_CM_, T_MM_, and T_EM_ can all differentiate into skin T_RM_ cells. Among these populations, T_CM_ emerged as the most efficient precursor, generating the largest and most diverse pool of long-lived T_RM_ cells. Notably, T_CM_-derived T_RM_ were enriched for FOXP3-expressing cells, suggesting that circulating T_CM_ may also contribute to the development of skin-resident regulatory T (regT_RM_) cells (14).

### Origin of regulatory T_RM_ cells

In contrast to the proposed T_CM_-based model discussed above, CD45RO^+^ antigen-experienced memory Treg cells are detectable even in human fetal skin (15, 16), suggesting that the establishment of skin-resident Treg cells begins early in human development. Although direct evidence for their developmental origin and long-term persistence in humans remains limited, murine studies provide supportive mechanistic insights. Using neonatal mouse models and inducible Treg depletion systems, Scharschmidt et al. demonstrated that a wave of regulatory T cells enters the skin during early life and is required for establishing tolerance to commensal microbes. Consistent with this finding, genetic fate-mapping studies showed that many skin Treg cells generated during this neonatal period persist into adulthood with minimal replacement (17, 18). Together, these studies suggest that skin Treg cells are largely established early in life from thymus-derived Treg populations in mice, and possibly also in humans.

However, recent work by Zhang et al. demonstrated in mice that peripherally induced RORγt^+^ regT_RM_ cells contribute to allergen-specific immunotherapy–induced immune tolerance. Notably, an expansion of RORγt^+^ Treg cells was also observed in the skin of human responders to immunotherapy, raising the possibility that peripherally induced Treg populations may contribute to human regT_RM_ compartments as well (19).

Therefore, although murine studies have largely emphasized neonatal thymus-derived Treg cells as the principal source of skin regT_RM_ cells, the relative contribution of circulating memory T-cell populations and peripherally induced Treg cells remains incompletely understood. Given the substantial differences between murine and human skin immunity, whether the developmental hierarchy observed in mice directly applies to human skin regT_RM_ cells remains to be determined.

## Resident memory T cells in normal human epidermis

### Composition and phenotypic characteristics

#### Epidermal conventional T_RM_ cells

The normal human epidermis harbors a population of T_RM_ cells (2). To compare resident T-cell populations in the epidermis and dermis, normal human skin was separated into epidermal and papillary dermal compartments using Dispase II and cultured *ex vivo*, after which emigrating cells were analyzed by flow cytometry (20). Using this approach, we estimated that the epidermis contains approximately 1 × 10^4^ CD3^+^ T cells/cm², whereas the papillary dermis contains approximately 2 × 10^5^ CD3^+^ T cells/cm². Although direct quantitative comparison between these compartments is complicated by marked differences in tissue thickness and cellular composition, substantial T-cell populations were identified in both. Analysis of memory T-cell subsets revealed that the epidermal T_RM_ compartment was composed predominantly of CD8^+^ T_RM_ cells (approximately 80%) and a smaller proportion of CD4^+^ T_RM_ cells (approximately 20%). In contrast, dermal T_RM_ cells were enriched for CD4^+^ populations, consisting of approximately 40% CD8^+^ T_RM_ cells and 60% CD4^+^ T_RM_ cells (20). This compartment-specific distribution suggests that epidermal and dermal T_RM_ cells may fulfill distinct immunological functions within human skin.

Regardless of the method used for analysis, nearly all skin T_RM_ cells expressed CD69, supporting its utility as a canonical marker of tissue residency in human skin (2, 20–22). Importantly, accumulating evidence from murine studies indicates that CD69 is not merely a marker of CD4^+^ and CD8^+^ T_RM_ cells but also a functional regulator of tissue residency, promoting long-term retention within peripheral tissues by suppressing S1PR1-dependent tissue egress (23–25).

CD103 expression was comparable between CD4^+^ and CD8^+^ T_RM_ cells in both the epidermis and dermis. However, approximately 70% of epidermal T_RM_ cells expressed CD103, compared with only 30% of dermal T_RM_ cells, suggesting preferential enrichment of CD103^+^ T_RM_ cells within the epidermis (2, 20). The predominance of CD103^+^ T_RM_ cells in the epidermis supports the concept that the epidermis functions as a specialized T_RM_ niche.

Another molecule implicated in epidermal T_RM_ retention is CD49a. CD49a constitutes the α-subunit of the α1β1 integrin receptor and binds to collagen IV, which is enriched within the basement membrane separating the epidermis and dermis. Although the human epidermis is characterized by an enrichment of CD8^+^ T_RM_ cells and a higher frequency of CD103 expression compared with the dermis, Cheuk et al. further identified a distinct population of CD69^+^CD103^+^CD49a^+^ T_RM_ cells that was almost exclusively localized to the epidermis. Notably, CD49a expression was largely confined to CD8^+^ T_RM_ cells, defining a cytotoxic T_RM_ subset characterized by IFN-γ production and the inducible expression of perforin and granzyme B, in contrast to CD49a⁻ T_RM_ cells, which preferentially exhibited an IL-17–associated program (21). Recent work has provided mechanistic insight into the differentiation of human cytotoxic CD49a^+^ epidermal T_RM_ cells. Using chromatin profiling and gene-editing approaches, Zitti et al. demonstrated that RUNX2 and RUNX3 cooperatively promote CD49a expression and cytotoxic programming in human epidermal CD8^+^ T_RM_ cells, supporting their role in immunosurveillance of infected and transformed cells (26). To investigate the functional role of CD49a, Bromley et al. employed a murine HSV skin infection model. Although CD49a was dispensable for skin entry and T_RM_ formation, it promoted the long-term persistence and protective function of epidermal T_RM_ cells. CD49a deficiency resulted in reduced T_RM_ numbers, impaired dendritic morphology, and diminished IFN-γ production upon antigen re-exposure, suggesting that CD49a primarily supports the maintenance and effector function of epidermal T_RM_ cells within the collagen IV-rich epidermal niche (27).

Taken together, the normal human epidermal T_RM_ compartment is composed predominantly of CD8^+^ T_RM_ cells (approximately 80%), with CD4^+^ T_RM_ cells accounting for the remaining 20%. Approximately 70% of epidermal CD8^+^ and CD4^+^ T_RM_ cells express CD103, while CD49a is preferentially expressed by a subset of CD69^+^CD103^+^ CD8^+^ T_RM_ cells. Importantly, CD69, CD103, and CD49a function not only as defining markers but also as key regulators of tissue residency, mediating tissue retention, epidermal localization, and long-term persistence, respectively.

#### Epidermal regulatory T_RM_ cells

Before the concept of T_RM_ cells was formally established, Treg cells had already been recognized to reside in human peripheral tissues, including the skin, under steady-state conditions (28, 29). Human epidermal and dermal Treg cells appear to occupy distinct microenvironmental niches and rely on different mechanisms for their maintenance. In the human dermis, Treg-cell proliferation is supported by dermal fibroblasts and IL-15 through cell–cell contact in the absence of classical antigen presentation or costimulation, and their suppressive activity is likewise mediated through direct cellular interactions rather than IL-10 or TGF-β production (28). In contrast, human epidermal Treg-cell proliferation is promoted by epidermal Langerhans cells (LCs), the professional antigen-presenting cells of the epidermis, highlighting a tolerogenic role for LCs in the steady-state epidermis (30). Epidermal regT_RM_ cells suppress the proliferation of autologous skin T_RM_ cells through a cell contact-dependent mechanism requiring MHC class II and CD80/CD86, suggesting the involvement of antigen presentation (30, 31). Consistent with these observations, both Foxp3^+^ and Ki-67^+^ cells are significantly enriched among epidermal compared with dermal T cells, indicating active *in situ* proliferation of epidermal regT_RM_ cells (30). Together, these findings suggest that epidermal and dermal Tregs are maintained by distinct cellular networks and may play complementary roles in regulating local skin immunity.

Analysis of isolated human epidermal and epithelial compartments further refined the phenotype of regT_RM_ cells. Approximately 10–15% of epidermal CD4^+^ T_RM_ cells expressed FOXP3, indicating the presence of a substantial regulatory compartment within the epidermal T-cell pool. In contrast to conventional epidermal T_RM_ cells, which are frequently CD103^+^, epidermal regT_RM_ cells were found to reside predominantly within the CD4^+^CD103⁻ population and exhibited high FOXP3 demethylation, supporting their bona fide regulatory lineage. These cells expressed multiple Treg-associated molecules, including CD25, CTLA-4, PD-1, ICOS, TIGIT, and CD39, produced relatively low levels of inflammatory cytokines, and efficiently suppressed the proliferation of neighboring epidermal T_RM_ cells. Importantly, CD4^+^CD103⁻FOXP3^+^ regT_RM_ cells were consistently identified across multiple human epithelia, suggesting that this population represents a conserved epithelial regulatory network (31). Interestingly, although epidermal Treg cells have been proposed to be maintained by LCs and preferentially localized around hair follicles, the frequency of CD4^+^CD103⁻FOXP3^+^ regT_RM_ cells was significantly lower in the scrotal epidermis despite its high hair follicle density. Conversely, comparable populations were identified in the vaginal epithelium, glans penis, and urethral epithelium, tissues that lack hair follicles and contain few or no LCs (31). These observations suggest that the generation and maintenance of epithelial regT_RM_ cells are not solely dependent on hair follicles or LC-mediated regulation. Collectively, these findings indicate that epithelial immune homeostasis is maintained by a specialized compartment of regT_RM_ cells that is conserved across diverse human epithelial tissues, although the mechanisms governing their development and persistence remain largely unknown.

#### Summary

**Figure 1:**
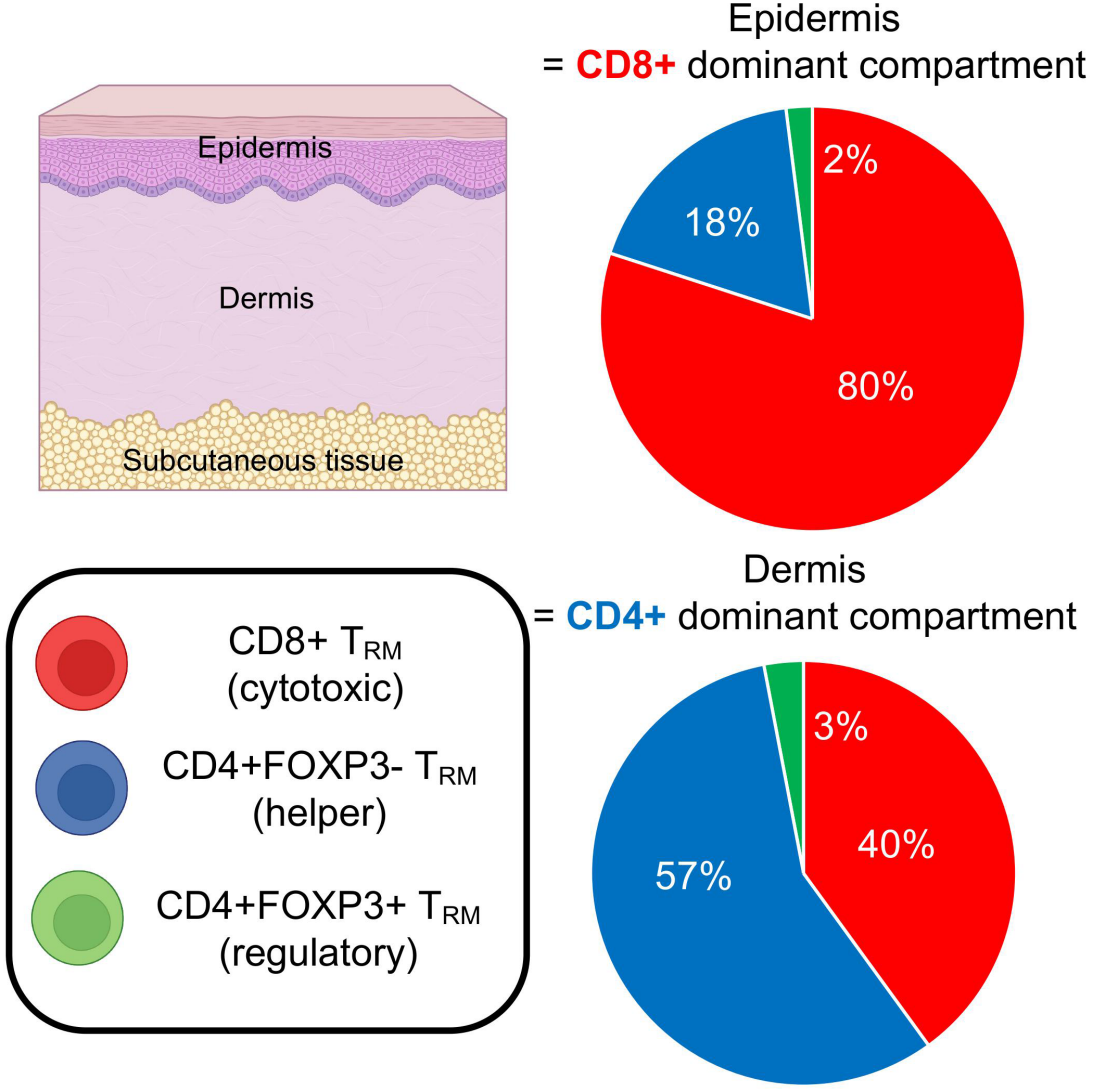
Distinct composition of epidermal and dermal resident memory T-cell compartments in normal human skin. Schematic illustration of the distribution of T_RM_ subsets in normal human skin. The epidermis is predominantly populated by CD8^+^ T_RM_ cells (approximately 80%), whereas CD4^+^Foxp3⁻ T_RM_ cells and CD4^+^Foxp3^+^ regT_RM_ cells account for approximately 18% and 2% of epidermal T cells, respectively. In contrast, the dermal T_RM_ compartment is enriched for CD4^+^Foxp3⁻ T_RM_ cells (approximately 57%), with CD8^+^ T_RM_ cells and CD4^+^Foxp3^+^ regT_RM_ cells comprising approximately 40% and 3%, respectively. Created with BioRender.com.

These studies have established that the normal human epidermis harbors a highly organized resident T-cell network composed of approximately 80% CD8^+^ T_RM_ cells, 18% CD4^+^Foxp3⁻ T_RM_ cells, and 2% CD4^+^Foxp3^+^ regT_RM_ cells, highlighting the coordinated contributions of cytotoxic, helper, and regulatory T-cell populations to epidermal immune homeostasis. In contrast, the dermal T_RM_ compartment exhibits a markedly different composition, consisting of approximately 40% CD8^+^ T_RM_ cells, 57% CD4^+^Foxp3⁻ T_RM_ cells, and 3% CD4^+^Foxp3^+^ regT_RM_ cells (32) (**Figure 1**).

As reference, it should be noted that most studies discussed above examined the epidermis together with the papillary/subpapillary dermis because removal of the reticular dermis is required for efficient separation of the epidermis from the underlying dermis. Analyses of the reticular dermis revealed that both convT_RM_ and regT_RM_ cells are also present in deeper dermal layers. Although their phenotypic characteristics, including CD69 and CD103 expression, as well as their cytokine-producing capacities, were largely comparable between the upper and lower reticular dermis, both convT_RM_ and regT_RM_ cells were significantly more abundant in the upper reticular dermis. These findings suggest that, unlike the marked compartmentalization observed between the epidermis and dermis, resident T-cell populations are relatively homogeneous throughout the reticular dermis, with quantitative rather than qualitative differences between dermal layers (33).

### Functional heterogeneity of epidermal T_RM_

More recently, CD101 has emerged as a novel marker that further refines the phenotypic and functional heterogeneity of epidermal T_RM_ cells. Although CD101 has been identified as a core signature of human CD8^+^ T_RM_ cells across multiple tissues (34), its ligand remains unknown and its precise biological function has yet to be fully elucidated. Nevertheless, accumulating evidence suggests that CD101 may play an immunoregulatory role. Genetic studies have shown that individuals carrying CD101 variants exhibit enhanced pro-inflammatory T-cell responses and impaired Treg-suppressive activity, indicating that CD101 contributes to the maintenance of immune homeostasis (35).

**Figure 2:**
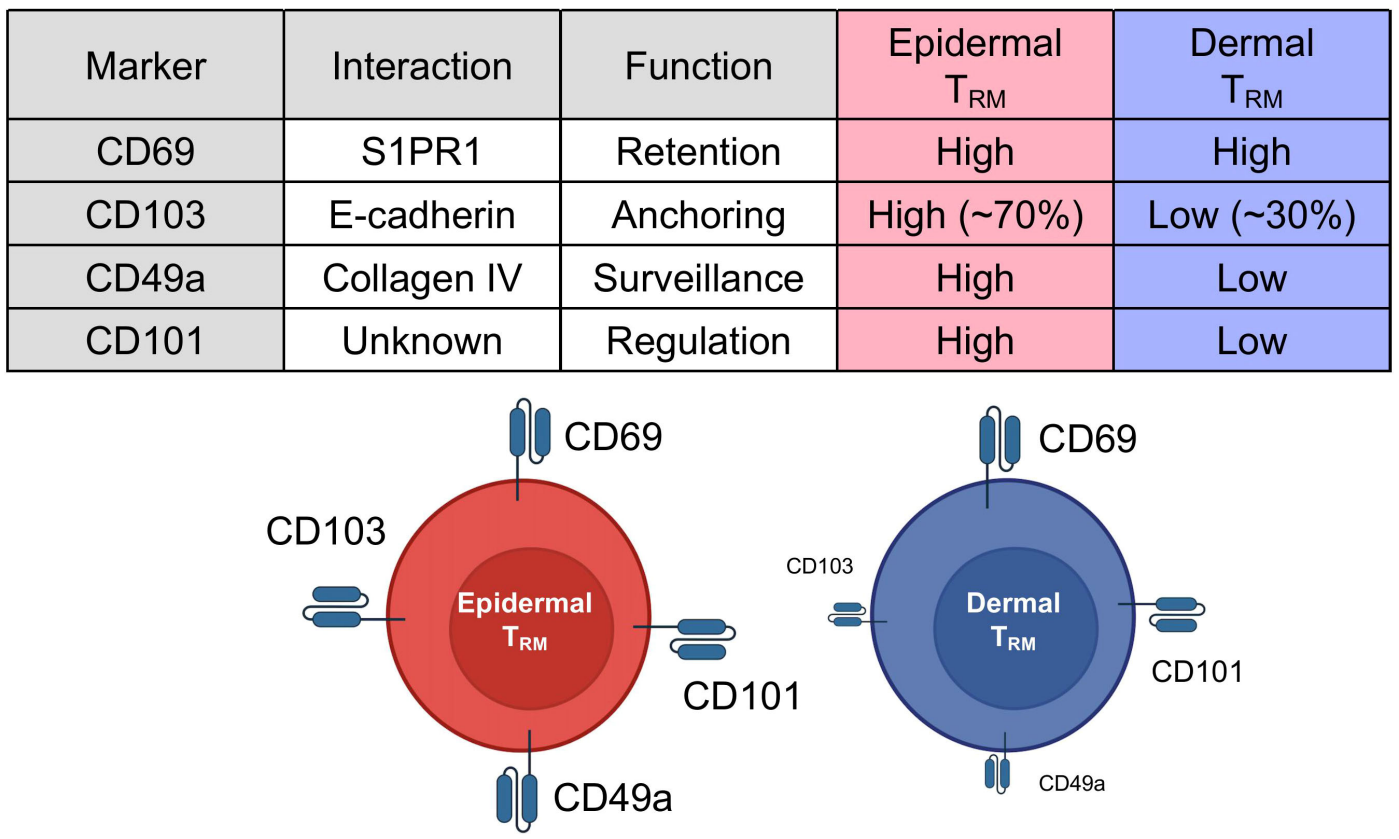
Phenotypic characteristics of epidermal and dermal T_RM_ cells. Comparison of representative residency-associated markers expressed by epidermal and dermal T_RM_ cells. CD69 promotes tissue retention through antagonism of sphingosine-1-phosphate receptor 1 (S1PR1) and is highly expressed in both epidermal and dermal T_RM_ cells. CD103 binds E-cadherin and mediates epithelial anchoring, with markedly higher expression in epidermal T_RM_ cells. CD49a interacts with collagen IV within the basement membrane and contributes to tissue surveillance and positioning. CD101, a marker associated with terminal differentiation and immune regulation, is preferentially expressed by epidermal T_RM_ cells. Created with BioRender.com.

In normal human skin, CD101 expression is preferentially enriched in epidermal T_RM_ cells compared with their dermal counterparts and is particularly abundant in both epidermal regT_RM_ cells and cytotoxic CD8^+^ T_RM_ cells. Approximately 90% of epidermal regT_RM_ cells express CD101, and FOXP3 expression is largely confined to the CD4^+^CD103⁻CD101^+^ population (32), further refining the phenotypic definition of epidermal regT_RM_ cells beyond the previously recognized CD4^+^CD103⁻ phenotype (31). In parallel, CD101^+^ CD8^+^ T_RM_ cells preferentially produce IFN-γ and TNF-α, whereas CD101⁻ CD8^+^ T_RM_ cells are enriched for IL-17A production. Moreover, CD101 expression substantially overlaps with CD49a expression, and CD8^+^CD69^+^CD103^+^CD49a^+^CD101^+^ T_RM_ cells represent the most potent IFN-γ-producing epidermal T_RM_ subset identified to date (32). Thus, incorporation of CD101 further refines the identification of cytotoxic epidermal T_RM_ cells beyond the previously established CD69^+^CD103^+^CD49a^+^ phenotype (21) (**Figure 2**).

Interestingly, recent studies in non-cutaneous tissues have shown that CD101 is preferentially expressed by functionally stable Treg cells possessing Treg-specific epigenetic signatures and durable suppressive activity, suggesting that CD101 may mark highly specialized regulatory T-cell populations rather than simply serving as a phenotypic marker (36). Together with the observation that CD101 genetic variation is associated with reduced Treg-suppressive function, these findings raise the possibility that CD101 itself may contribute to the maintenance of regulatory programs in T_RM_ cells. Collectively, these findings suggest that CD101 serves as a useful marker for both regulatory and cytotoxic epidermal T_RM_ populations, although whether CD101 directly contributes to tissue residency, immune regulation, or effector function remains to be determined.

## Dynamics of human skin-resident memory T cells

### Entry

Although the developmental pathways leading to epidermal T_RM_-cell formation are becoming increasingly understood, the anatomical route by which circulating precursor cells enter the epidermis remains poorly defined, particularly in humans. To date, the most informative study has come from a murine model. Using adoptive transfer of splenic T cells into lymphopenic mice, it was demonstrated that CD4^+^ and CD8^+^ T_RM_ precursors enter the epidermis through distinct pathways. CD4^+^ T cells first accumulated within the dermis and around hair follicles before appearing in the epidermis, whereas CD8^+^ T cells were detected directly within the interfollicular epidermis and subsequently concentrated around hair follicles (37). These observations suggest that hair follicles may function as specialized niches that facilitate epidermal entry and establishment of skin T_RM_ cells. Hair follicle keratinocytes were shown to produce IL-7 and IL-15, cytokines required for the maintenance of epidermotropic CD4^+^ and CD8^+^ T_RM_ cells, respectively (37). Although comparable analyses have not yet been performed in human skin, these findings provide a framework for understanding how circulating precursor cells may gain access to the epidermal compartment and subsequently differentiate into epidermal T_RM_ cells.

### Differentiation and persistence

One of the defining features of human skin T_RM_ cells is their extraordinary longevity. Evidence from patients undergoing allogeneic hematopoietic stem cell transplantation demonstrated that host-derived skin T_RM_ clones can persist locally for at least a decade despite near-complete replacement of circulating T cells by donor-derived cells. Importantly, these long-lived T_RM_ cells remain functionally competent and retain the capacity to produce effector cytokines. Single-cell transcriptomic analyses revealed sustained expression of tissue-retention molecules and low expression of tissue-egress programs, supporting stable tissue residency. In addition, longitudinal T-cell receptor sequencing provided evidence that persisting skin T_RM_ clones can undergo local proliferation, indicating that long-term maintenance is achieved not only through survival but also through self-renewal within the tissue. These findings establish human skin T_RM_ cells as highly durable immune populations capable of maintaining local immune memory for many years (38). Consistent with the concept of long-term local persistence, intravital two-photon microscopy in murine skin demonstrated that epidermal CD8^+^ T_RM_ cells exhibit slow local migration while remaining largely confined to the site of their initial establishment, suggesting that local tissue microenvironments contribute to sustained tissue residency (39).

Mechanistic studies in murine skin first demonstrated that the development and long-term maintenance of CD103^+^ T_RM_ cells require both TGF-β and IL-15 signaling. TGF-β signaling was essential for CD103 induction and T_RM_ formation, whereas IL-15 supported T_RM_ survival within peripheral tissues (10). Consistent with these findings, Watanabe et al. subsequently demonstrated in a human skin xenograft model that keratinocyte contact induces CD103 expression on infiltrating T cells through a TGF-β-dependent mechanism, thereby validating in humans a mechanism that had originally been identified in murine T_RM_ models and providing direct evidence that local epidermal cues promote T_RM_ differentiation and retention in human skin (2). Furthermore, Hirai et al. showed in murine models that keratinocyte-expressed integrins αvβ6 and αvβ8 activate latent TGF-β within the epidermal niche and are indispensable for the maintenance of epidermal CD103^+^ T_RM_ cells, establishing a key mechanism that supports long-term epidermal T_RM_ residency (40). Mackay et al. subsequently demonstrated that TGF-β and IL-15 are mechanistically linked through regulation of the T-box transcription factors Eomes and T-bet. TGF-β promotes T_RM_ differentiation by suppressing Eomes and T-bet expression and inducing CD103, whereas residual T-bet expression maintains CD122 expression and IL-15 responsiveness, thereby supporting long-term survival of CD103^+^ T_RM_ cells. Together, these findings provide a mechanistic framework linking epidermal TGF-β signaling to both T_RM_ differentiation and persistence (41).

### Patrol and egress

Although T_RM_ cells have traditionally been regarded as sessile populations that permanently reside within peripheral tissues, recent studies have revealed that human skin T_RM_ cells exhibit unexpectedly dynamic behavior. Their dynamics can be broadly categorized into local tissue patrol within the skin and inter-tissue migration between distinct skin sites.

Within the human epidermis, CD8^+^ T_RM_ cells actively patrol the basal layer rather than remaining stationary. Real-time imaging demonstrated that epidermal T_RM_ cells continuously migrate along the dermal–epidermal junction in close proximity to the basement membrane, forming a dynamic surveillance network among basal keratinocytes. These cells frequently move beneath relatively sessile LCs, suggesting a division of labor in which motile T_RM_ cells perform active immune surveillance while LCs serve as resident antigen-presenting sentinels. Importantly, both CD103^+^ and CD103⁻ epidermal T_RM_ cells exhibit comparable migratory activity, indicating that tissue patrol is a fundamental property of epidermal T_RM_ cells regardless of CD103 expression (22).

In the human dermis, T_RM_ cells display distinct migratory patterns influenced by local tissue architecture. Dermal T_RM_ cells migrate through both collagen-rich and collagen-poor regions and frequently move along dermal vascular structures. The observation that T_RM_ cells exhibit faster movement in collagen-poor areas suggests that extracellular matrix composition contributes to the regulation of dermal T_RM_ motility. Furthermore, some T_RM_ cells were observed to move across the dermal–epidermal junction, indicating that epidermal and dermal T_RM_ populations may not represent completely isolated compartments (22).

Beyond local tissue patrol, a subset of human skin T_RM_ cells possesses the capacity to exit the skin and recirculate. Human CD4^+^CD103^+^ skin T_RM_ cells can downregulate CD69, leave the skin, enter the blood and lymphatic circulation, and subsequently reseed distant skin sites, where they reacquire a resident phenotype. These findings challenge the classical view of T_RM_ cells as permanently tissue-confined populations and suggest that at least a subset of skin T_RM_ cells exists in a dynamic equilibrium between tissue residency and migration (42). Supporting this concept, Strobl et al. identified a population of circulating T_RM_-like cells (cT_RM_) in humans that retained phenotypic and transcriptional features of skin T_RM_ cells and could be traced back to a cutaneous origin. Importantly, these cells were enriched during active graft-versus-host disease and produced pro-inflammatory Th2- and Th17-associated cytokines, suggesting that skin-derived T_RM_ cells may contribute to inflammatory responses beyond their original tissue of residence. These observations indicate that T_RM_ egress is not merely a mechanism for redistributing immune memory but may also represent a pathway through which tissue-resident immune responses influence systemic inflammation (43).

Collectively, current evidence indicates that human skin T_RM_ cells exhibit multiple layers of mobility: continuous surveillance within the epidermis and dermis, limited trafficking across the dermal–epidermal interface, and, in some subsets, recirculation through the blood and lymphatic system with the potential to influence inflammatory responses at distant tissue sites.

## Maintenance and regulation of epidermal T_RM_ cells

### Aging

Aging profoundly affects circulating T-cell immunity, leading to reduced naïve T-cell numbers, diminished T-cell receptor (TCR) diversity, and impaired pathogen-specific immune responses. In contrast, human skin T_RM_ cells are remarkably preserved with age. Epidermal T-cell density does not decline and may even increase in elderly individuals, accompanied by enrichment of CD49a^+^ cytotoxic CD8^+^ T_RM_ cells. Despite these changes, epidermal T_RM_ cells maintain cytokine-producing capacity and TCR diversity (44). Dermal T_RM_ populations are likewise relatively stable during aging, exhibiting preserved cellularity and functional competence (44). Recent evidence from a comprehensive pan-tissue analysis of human memory T cells further supports this concept. Using retrospective radiocarbon birth dating combined with transcriptomic and epigenetic profiling, Lam et al. demonstrated that T_RM_ cells across multiple tissues maintain site-specific resident phenotypes throughout life and largely avoid the immunosenescent features observed in circulating effector-memory T cells. These findings suggest that tissue residency may provide a unique framework for long-term immune maintenance and help explain the remarkable preservation of skin T_RM_ cells during aging (45). Collectively, these findings suggest that skin T_RM_ cells, particularly epidermal T_RM_ cells, constitute long-lived and resilient immune populations that may provide durable tissue protection throughout life.

### Langerhans cells

**Figure 3:**
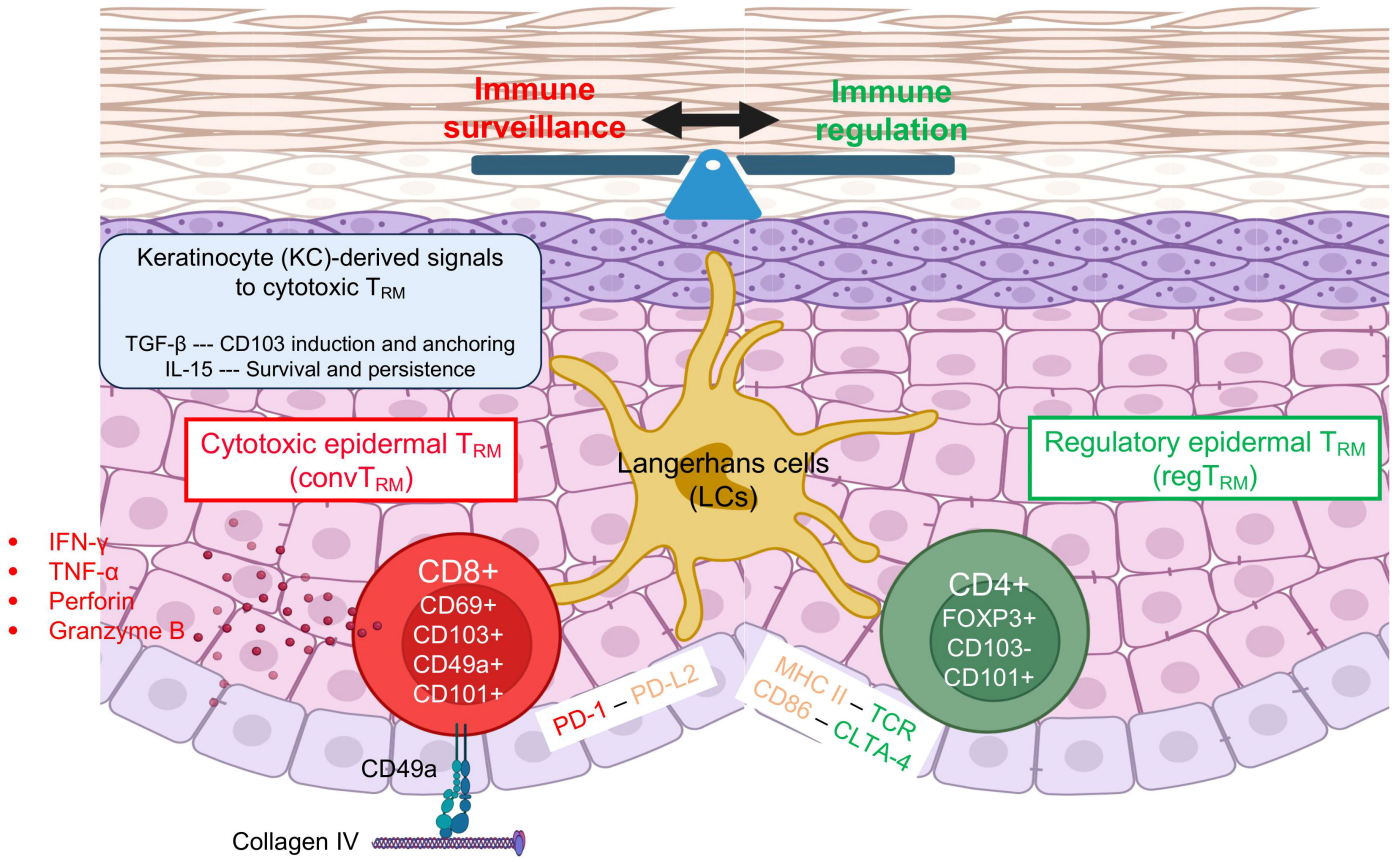
Proposed epidermal immune ecosystem maintained by interactions among keratinocytes, Langerhans cells, cytotoxic T_RM_ cells, and regulatory T_RM_ cells. Schematic model illustrating the coordinated cellular interactions that maintain epidermal immune homeostasis. Cytotoxic epidermal T_RM_ cells provide local immune surveillance through the production of effector molecules, including IFN-γ, TNF-α, perforin, and granzyme B. Regulatory epidermal T_RM_ cells suppress excessive immune activation and contribute to tissue homeostasis. Langerhans cells (LCs) interact with convT_RM_ cells through PD-L2–PD-1 signaling and with regT_RM_ cells through MHC class II–TCR and CD86–CTLA-4 pathways. Keratinocytes support epidermal T_RM_ maintenance by providing TGF-β, which promotes CD103 expression and epithelial anchoring, and IL-15, which supports T_RM_ survival and long-term persistence. In addition, CD49a-mediated interactions with collagen IV contribute to T_RM_ localization within the epidermal niche. Created with BioRender.com.

In both humans and mice, LCs have been implicated in the proliferation and activation of skin-resident regulatory T cells, suggesting that LC–Treg interactions represent a conserved mechanism for maintaining epidermal immune homeostasis (30, 46, 47). Recent analyses of normal human epidermis have further demonstrated that LCs directly interact not only with Treg cells but also with conventional T_RM_ cells. In normal human skin, epidermal LCs physically contact both CD4^+^ and CD8^+^ CD103^+^ T_RM_ cells, and the frequency of these interactions increases with aging, paralleling the age-associated accumulation of epidermal T_RM_ cells. LCs engaged with T_RM_ cells exhibit high expression of MHC class II, CD86, and PD-L2, whereas the interacting T_RM_ cells preferentially express PD-1, suggesting the existence of a local tolerogenic axis that restrains excessive T_RM_-cell activation within the steady-state epidermis. Importantly, environmental insults such as UVB irradiation and contact allergens disrupt this interaction by downregulating MHC class II and CD86 expression on epidermal LCs. Furthermore, PD-1 blockade therapy markedly reduces LC–T_RM_ contact in normal human epidermis, supporting the notion that LC-mediated regulation contributes to epidermal immune tolerance. In addition, LCs also directly interact with epidermal regT_RM_ cells. LC-contacting CD4^+^ T cells are enriched for CTLA-4 and FOXP3 expression, and most CTLA-4^+^ epidermal T_RM_ cells exhibit a regulatory phenotype (48). In summary, these findings suggest that LCs function as local regulators of both convT_RM_ cells and regT_RM_ cells in the human epidermis through direct cell–cell interactions, thereby contributing to the maintenance of epidermal immune homeostasis and peripheral tolerance (**Figure 3**).

## Pathological relevance of human epidermal T_RM_ cells

The contribution of human skin T_RM_ cells, including epidermal T_RM_ populations, to the pathogenesis of various skin diseases has been extensively studied. Their roles are particularly well established in inflammatory skin disorders such as psoriasis and atopic dermatitis, as well as in cutaneous T-cell lymphomas, including mycosis fungoides. Because these topics have been comprehensively covered in several excellent reviews, readers are referred to those reviews for more detailed discussions (1, 49–51).

### HIV infection

Although direct evidence for the pathological role of human epidermal CD4^+^ T_RM_ cells remains limited, studies of mucosal CD4^+^ T_RM_ cells in HIV infection provide valuable insights into the potential biological and pathological significance of epidermal CD4^+^ T_RM_ cells. Exposure of mucosal surfaces to mucus containing HIV virions initiates infection at the epithelial barrier. Traditionally, mucosal epithelial LCs have been considered the primary target cells for HIV. HIV infects LCs through CD4 and CCR5, after which infected LCs migrate to draining lymph nodes and transfer virions to CD4^+^ T cells, thereby establishing lifelong infection (52, 53). However, the identification of CD4^+^ T_RM_ cells within the human epidermis and mucosal epithelia has expanded our understanding of the cellular targets of HIV infection. Because CD4^+^ T_RM_ cells are also susceptible to HIV infection, their potential contribution to viral persistence has attracted increasing attention. Although T_RM_ cells are generally regarded as long-lived residents of peripheral tissues, accumulating evidence, as discussed above, indicates that they can downregulate CD69 and egress from tissues, raising the possibility that they may contribute not only to local viral persistence but also to systemic dissemination. Indeed, in human cervical mucosa, CD4^+^ T_RM_ cells preferentially support HIV infection and harbor substantially higher levels of viral DNA and RNA, even during long-term suppressive antiretroviral therapy (54). These findings identify CD4^+^ T_RM_ cells as a viral reservoir and suggest that the longevity and self-renewal capacity of T_RM_ cells, which are normally beneficial for tissue immunity, may inadvertently facilitate viral persistence. These observations suggest that epidermal and mucosal CD4^+^ T_RM_ cells may represent a double-edged sword: while they provide durable local immune protection, they may also constitute a persistent cellular niche for HIV infection.

### Fixed drug eruption

Fixed drug eruption (FDE) is a distinctive form of cutaneous adverse drug reaction characterized by the recurrence of well-demarcated erythematous or violaceous lesions at the same anatomical sites upon re-exposure to the causative drug. Following resolution, the lesions typically leave residual hyperpigmentation, reflecting the persistence of local immunological memory within the affected skin. As mentioned above, even before the concept of T_RM_ cells was established, it was recognized that long-lived CD8^+^ T cells persist at the dermal–epidermal junction of resolved FDE lesions. These resident T cells remain poised for rapid reactivation upon drug re-exposure and are considered central mediators of lesion recurrence, making FDE one of the earliest human disease models demonstrating the biological principles that are now attributed to T_RM_ cells (4, 55). More recently, Matsumura et al. extended this concept by demonstrating that pathogenic CD49a^+^ cytotoxic T_RM_ (cT_RM_) cells in recurrent FDE lesions exhibit features of phenotypic plasticity. Through TCR repertoire analysis, they identified a population of CCR7^+^CD49a^+^ T cells with characteristics intermediate between T_RM_ and T_CM_ cells and showed substantial TCR clonal overlap between lesional cT_RM_ cells and these T_CM_-like cells (56). These findings suggest that resident pathogenic T_RM_ cells can acquire migratory properties while maintaining clonal identity, providing a potential explanation for the expansion and recurrence of FDE lesions. Thus, FDE serves not only as a prototypic T_RM_-mediated disease but also as a valuable human model for studying T_RM_ plasticity and the generation of ex-T_RM_ populations.

### Vitiligo

Vitiligo is a common acquired depigmenting disorder characterized by the selective destruction of melanocytes, resulting in well-demarcated white patches of skin. Although its pathogenesis is multifactorial, autoreactive CD8^+^ T cells are considered the principal mediators of melanocyte destruction through the production of IFN-γ and cytotoxic molecules. The characteristic tendency of vitiligo to recur at previously affected sites has led to increasing interest in the role of skin T_RM_ cells in disease persistence and relapse (57). In support of this concept, Boniface et al. demonstrated that both active and stable vitiligo lesions are enriched with CD69^+^CD103^+^ CD8^+^ T_RM_ cells expressing CXCR3. Importantly, CD103^+^ CD8^+^ T_RM_ cells were predominantly localized within the epidermis, where melanocytes reside, supporting the concept that epidermal T_RM_ cells contribute directly to local disease recurrence and persistence (58). Using a mouse model of vitiligo, Richmond et al. further demonstrated that vitiligo lesions harbor a population of CD8^+^ T_RM_ cells expressing CD122, a component of the IL-15 receptor, indicating their dependence on IL-15 signaling for long-term maintenance. The authors showed that blockade of IL-15 signaling resulted in depletion of pathogenic T_RM_ cells and durable repigmentation. Consistent with these findings, human vitiligo skin contained abundant CD69^+^CD103^+^ CD8^+^ T_RM_ cells, further supporting the notion that T_RM_ cells contribute to disease persistence and represent a promising therapeutic target in vitiligo (59). These observations established vitiligo as one of the best-characterized human diseases in which pathogenic CD8^+^ T_RM_ cells are thought to maintain local immunological memory and drive disease relapse.

### Alopecia areata

Alopecia areata (AA) is an autoimmune hair loss disorder driven by IFN-γ-producing cytotoxic CD8^+^ T cells targeting hair follicles. Recent studies have identified increased numbers of CD69^+^CD103^+^ and CD8^+^CD103^+^ T_RM_ cells in chronic and treatment-refractory AA lesions. These T_RM_ cells are associated with local IL-15 expression and exhibit features consistent with long-term tissue persistence. Furthermore, JAK inhibitor therapy was accompanied by a reduction in lesional T_RM_ cells, suggesting that T_RM_ populations may contribute to disease chronicity and relapse (60–62).

## Discussion

**Figure 4:**
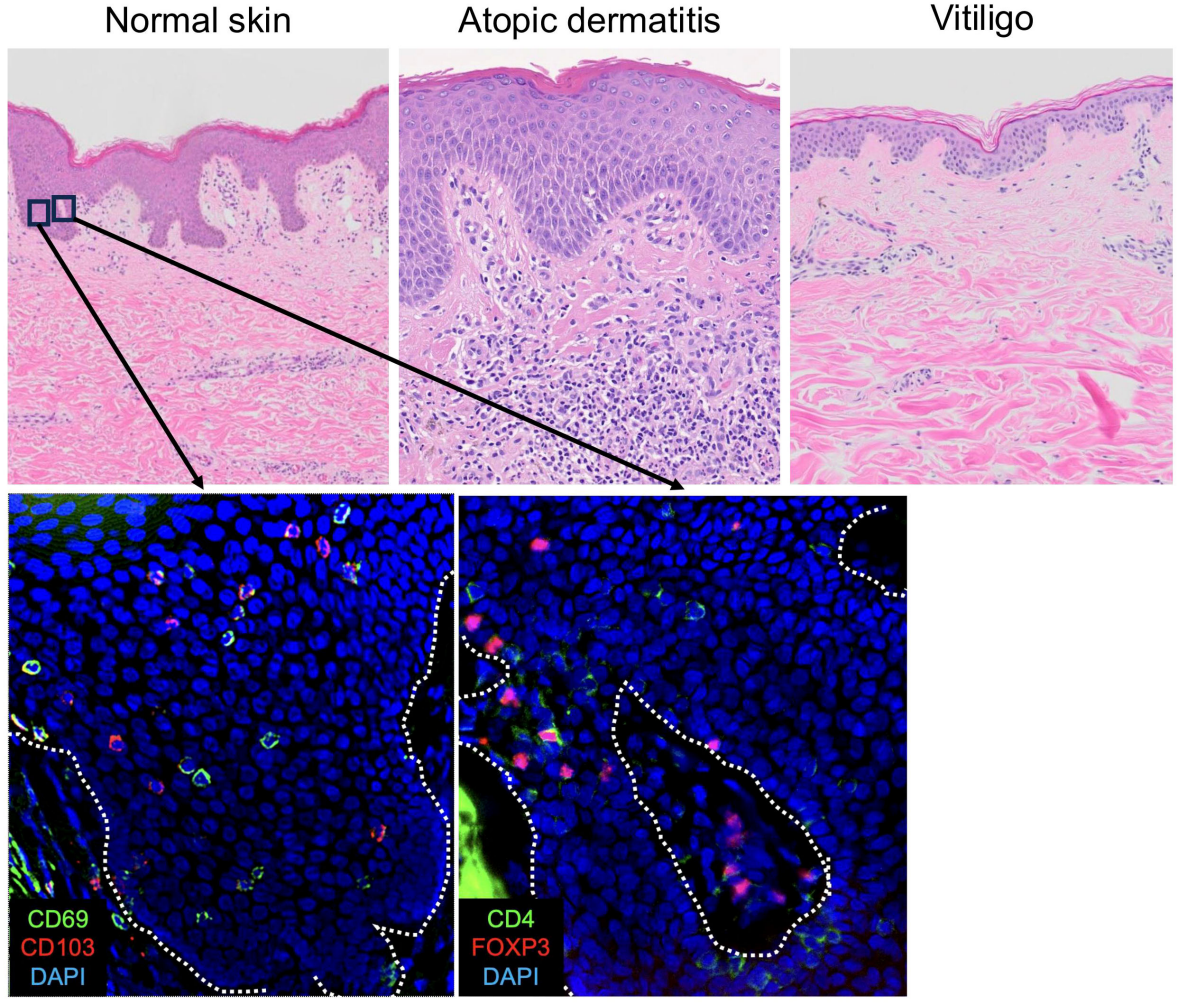
Limited visibility of epidermal immune cells on routine histopathology. Representative H&E sections of normal skin, atopic dermatitis, and vitiligo, together with representative immunofluorescence images of normal human epidermis showing CD69/CD103 and CD4/FOXP3 staining. While epidermal immune-cell populations such as T_RM_ cells, regulatory T_RM_ cells, and LCs are not readily identifiable on routine H&E staining, immunofluorescence clearly demonstrates the presence of organized immune-cell populations within the normal epidermis. The H&E images are original images from the authors’ archive. The immunofluorescence images are reproduced from our previous publications (Refs. 20 and 31), published under the Creative Commons Attribution (CC BY) license.

Traditionally, cutaneous inflammation has been viewed primarily as a process originating in the dermis and subsequently extending into the epidermis. This concept likely arose, at least in part, from routine histopathological observations. On hematoxylin and eosin staining, neither T_RM_ cells nor even LCs are readily visible within the epidermis. The epidermis is overwhelmingly composed of densely packed keratinocytes interconnected by strong cell–cell adhesion structures such as desmosomes, resulting in a cellular architecture that is substantially more compact than that of the dermis (63). Consequently, relatively sparse immune-cell populations are easily overlooked within the epidermal compartment. Although dermal inflammation undoubtedly influences epidermal immune responses, the recognition that the epidermis harbors abundant populations of T_RM_ cells raises the possibility that inflammatory responses may, at least in part, be autonomously initiated, amplified, and regulated within the epidermis itself. Indeed, in vitiligo, melanocyte destruction is thought to be mediated primarily by epidermal CD8^+^ T_RM_ cells despite the fact that inflammatory cell infiltration within the dermis is often minimal or even absent on routine histopathological examination. Such observations challenge the traditional dermis-centric view of cutaneous immunity and suggest that the epidermis should be regarded as an independent immunological compartment rather than merely a passive target of dermal inflammation (**Figure 4**).

Another notable feature of the normal human epidermis is its enrichment for CD69^+^CD103^+^ CD8^+^ T_RM_ cells, many of which express CD49a and CD101 and exhibit a cytotoxic, IFN-γ–producing phenotype. Thus, even under steady-state conditions, the epidermis appears to maintain a predominantly Tc1-oriented immune environment. Whether this reflects continuous exposure to environmental insults at the skin surface or represents an evolutionary adaptation for protection against diseases arising primarily within the epidermis remains unknown. In this regard, it will be important to investigate the abundance, phenotype, and functional properties of epidermal T_RM_ cells in diseases whose primary pathology is localized to the epidermis, including intraepidermal malignancies such as melanoma *in situ*, actinic keratosis, Bowen disease, and extramammary Paget disease; epidermotropic viral infections such as verruca vulgaris; and autoimmune disorders such as vitiligo and alopecia areata.

Importantly, the emerging picture of epidermal immunity extends beyond the biology of T_RM_ cells alone. The studies discussed in this review collectively suggest that epidermal immune homeostasis is maintained through a highly integrated cellular network composed of conventional T_RM_ cells, regulatory T_RM_ cells, LCs, and keratinocytes. Keratinocytes provide structural and immunological support for epidermal T_RM_ cells through the production of TGF-β, IL-7, and IL-15, thereby promoting tissue residency and long-term survival (2, 10, 37, 40). LCs directly interact with both conventional and regulatory T_RM_ populations and appear to function as local immune regulators through antigen presentation, co-stimulatory pathways, and PD-1–associated inhibitory signals (48). In parallel, epidermal regT_RM_ cells restrain excessive activation of neighboring conventional T_RM_ cells and contribute to the maintenance of local immune tolerance (30, 31). Thus, the epidermis should not be viewed simply as an epithelial barrier populated by resident lymphocytes, but rather as a highly organized immunological ecosystem in which epithelial cells and multiple resident immune-cell populations cooperate to balance immune surveillance and immune regulation. Recent advances in T_RM_ biology further support the view that tissue immunity is organized at the level of specialized microenvironmental niches rather than entire organs, highlighting the importance of local cellular interactions in shaping immune-cell behavior within the epidermis (64).

This concept may also help explain why many epidermis-centered diseases exhibit remarkable site specificity, chronicity, and recurrence. Disruption of the balance among conventional T_RM_ cells, regulatory T_RM_ cells, LCs, and keratinocytes may shift the epidermal immune ecosystem toward either excessive inflammation or impaired immune surveillance, thereby contributing to disease development. Future studies should therefore focus not only on individual epidermal cell populations but also on the cellular interactions that collectively govern epidermal immune homeostasis.

Although the epidermal immune ecosystem described above is based primarily on observations in the steady-state human epidermis, additional immune-cell populations may participate in epidermal immune responses during inflammation. Experimental studies have suggested that T_RM_ reactivation can influence broader immune networks beyond the epidermal compartment, although the relevance of these mechanisms to human skin disease remains incompletely understood (65–67).

## Conclusion and outlook

The discovery of epidermal T_RM_ cells has fundamentally advanced our understanding of human skin immunity. Accumulating evidence indicates that the epidermis harbors specialized populations of conventional and regulatory T_RM_ cells that cooperate with LCs and keratinocytes to maintain local immune homeostasis. Future studies integrating spatial biology, single-cell technologies, and *in situ* imaging will further elucidate how this epidermal immune network is organized in health and disrupted in disease (57). Such insights may facilitate the development of novel therapeutic strategies for epidermis-centered inflammatory, infectious, and neoplastic disorders.
